# “No‐one knows how to care for LGBT community like LGBT do”^1^: LGBTQIA+ experiences of COVID‐19 in the UK and Brazil

**DOI:** 10.1111/disa.12565

**Published:** 2022-10-30

**Authors:** Billy Tusker Haworth, Luan Carpes Barros Cassal, Tiago de Paula Muniz

**Affiliations:** ^1^ School of Geosciences University of Sydney Australia; ^2^ Humanitarian and Conflict Response Institute University of Manchester United Kingdom; ^3^ Manchester Institute of Education University of Manchester United Kingdom

**Keywords:** Brazil, COVID‐19, LGBTQIA+, marginality, resilience, United Kingdom

## Abstract

The coronavirus pandemic and responses have had uneven impacts on different segments of societies. We analysed experiences of LGBTQIA+ people during COVID‐19, based on interviews in the UK and Brazil in 2020. The UK and Brazil are instructive cases, with the same crisis impacting these two different social, cultural, economic, and political contexts. Pre‐existing marginalisation shaped COVID‐19 experiences in both contexts, influencing challenges faced, such as isolation or disruption to transgender healthcare, and coping strategies, including the important role of LGBTQIA+ volunteer and mutual‐aid groups. We argue that despite commonalities, there is no single LGBTQIA+ experience, and that disaster strategies will be ineffective until they recognise intersectionality and support the diversity of LGBTQIA+ populations. We conclude with a call to action for more inclusive disaster research, policy, and practice, which requires scrutinising dominant cisgender‐heteronormative structures that produce and reproduce LGBTQIA+ marginalisation.

### Introduction

The COVID-19 crisis, declared a global pandemic by the World Health Organisation (WHO) on March 11, 2020, presented serious threats and impacts on public health and safety, local and global economies, and international human rights (Bishop, 2020). The negative impacts of the virus and global governments' responses have been disproportionate for different population segments, including older adults (Lee and Miller, 2020), racial and ethnic minorities (Chin-Hong et al., 2020), and refugees and migrants (Kluge et al., 2020), among others. Vulnerability to crises, uneven risks, and coping capacities across populations are predominantly linked to pre-existing structural and systemic marginalisation, socioeconomic inequality, political decisions, ideologies, governance, societal roles, and power imbalances (Kelman, 2020).

Like other minority groups, lesbian, gay, bisexual, trans, queer, intersex, asexual and other minority gender/sexual identities (LGBTQIA+) experience heightened risk during crises, with the vulnerability (and resilience) of LGBTQIA+ populations born out of inequalities and marginalisation (Gorman-Murray et al., 2018). For example, LGBTQIA+ people experienced limited access to response and recovery support, such as food aid and shelter, following major disasters, including the 2010 Haiti earthquake and the 2011 Great East-Japan earthquake and tsunami, due to fears of discrimination and repression with lack of legal protections (Yamashita et al., 2017) or structural systems of relief that exclude people who don't align with cisgender, heterosexual family norms (Sauer and Podhora, 2013). During COVID-19, mental health decline, social isolation, substance misuse, financial stress,

This article has been accepted for publication and undergone full peer review but has not been through the copyediting, typesetting, pagination and proofreading process, which may lead to differences between this version and the Version of Record. Please cite this article as doi: 10.1111/disa.12565.

This article is protected by copyright. All rights reserved.

discrimination, elevated domestic violence risk, reduced access to health and support services, and more were reported as experienced by LGBTQIA+ people (LGBT Foundation, 2020; Bishop, 2020).

Despite growing awareness of LGBTQIA+ disaster experiences, these populations are often not planned for in crisis responses (Dominey-Howes et al., 2016; Reid and Ritholtz, 2020). Where LGBTQIA+ people have been included, diversity within LGBTQIA+ populations is often overlooked. This can contribute to further inequalities if those who are more visible, such as cisgender gay men (McKinnon et al., 2017), are seen to represent the views and experiences of all LGBTQIA+ people. Moreover, while identity labels (such as “gay” or “lesbian”) and collective terms (such as “LGBTQIA+”) can be useful in unifying against common oppressions, they can also homogenise and erase other instructive aspects of identity that shape diversity (and inequality) among LGBTQIA+ populations, such as race, nationality, or class (Anzaldúa, 1991).

Policies and activities aiming to address the disproportionate impacts of crises on ‘vulnerable’ groups such as LGBTQIA+ people must focus on the root causes of marginality, which requires understanding *intersectionality*. Arising from Black feminism activist scholarship (e.g., hooks, 1981), intersectionality theory describes how identities and inequalities intersect to shape experiences in everyday social, economic, and political life (Crenshaw, 1991; Ghabrial, 2019). “Intersectionality is not only about multiple identities but is about relationality, social context, power relations, complexity, social justice and inequalities” (Hopkins, 2019, p. 937). Crenshaw (1991) showed that to fully understand disadvantage and disempowerment, we must not consider factors of gender or race alone but recognise that these factors intersect to magnify and produce new modes of structural, political, and representational inequalities. Thus, marginality and vulnerabilities are not determined by single factors in isolation, but rather through complex intersections of multiple identity characteristics. Intersectionality challenges us to move beyond simplistic labels of ‘vulnerable’ and to see populations as diverse (Vickery, 2019). Disaster scholars have been calling for such nuance in understandings of LGBTQIA+ people’s crisis experiences and policies (Dominey-Howes et al., 2014), yet as COVID-19 has demonstrated, intersectional approaches remain absent in practice.

The multidimensional nature of vulnerability of gender and sexual minorities, and the urgency for States and other stakeholders to give visibility to and protect LGBTQIA+ people from suffering during COVID-19, was exemplified in a joint statement signed by 97 United Nations human rights experts (OHCHR, 2020). Another UN report concluded that pandemic responses around the world reproduced and intensified pre-existing social exclusion and discrimination patterns (Madrigal-Borloz, 2020), rather than providing targeted support. For example, same-sex couples in the Philippines were denied COVID-19 food aid because of local government’s cis-heteronormative views on what counts as a “family” (Ritholtz, 2020).

While investigating vulnerabilities remains important, it is also vital that we understand the resilience of LGBTQIA+ people and groups in navigating those challenges (Singh and McKleroy, 2011; Haworth, 2022). To date little research or policy attention has been given to coping strategies of LGBTQIA+ individuals and communities. Revelation of such experiences is particularly important when key aspects of community resilience, such as social connectedness (Haworth et al., 2018), were disrupted and eroded through COVID-19 social distancing, lockdowns, and other restrictions (Bishop, 2020).

This article is protected by copyright. All rights reserved.

Accepted Article

In this paper, we present and discuss the results of a study which investigated the experiences of LGBTQIA+ people in the United Kingdom (UK) and Brazil during the first wave of COVID-19 infections in 2020[2]. The study was guided by the following research questions:

1. How have COVID-19 and responses exposed, exacerbated, and/or created marginalisation and vulnerability of LGBTQIA+ people?
2. What coping strategies did LGBTQIA+ people utilise to live and adapt during the pandemic?
3. How can crisis responses and strategies be more inclusive of diverse LGBTQIA+ lives?

The UK and Brazil are instructive case studies as they represent different social, cultural, economic, and political contexts in which the same crisis is impacting[3]. This provides a rare opportunity to uncover common learnings and important differences in LGBTQIA+ responses to, and experiences of, humanitarian crises.

Next, we introduce the contexts of UK and Brazil as pertaining to COVID-19 in 2020, government responses, and LGBTQIA+ rights and experiences. We then present the study methods, before discussing key findings from interviews, arguing that there is no single LGBTQIA+ experience, and that policies and strategies will be ineffective until they recognise intersectionality and support the diversity of lived experiences among LGBTQIA+ populations, including existing coping and resilience capacities. We conclude with a call to action for more inclusive research, policy development, and disaster practices, which will require scrutinising the dominant cisheteronormative[4] structures that produce and reproduce marginalisation and vulnerability of gender and sexual minorities.

***The UK context***

There were nine confirmed cases of COVID-19 in the UK on February 24, rising to numbers in the thousands by late March (Pollock et al., 2020), as Europe became the global epicentre of the virus. Restrictions, such as business closures, were implemented in late March, and by April UK residents were advised to stay home to save lives and reduce pressure on the National Health Service (NHS), in what was the first of several national lockdowns (or 'stay at home' orders). Alternating periods of lockdown and relaxation of restrictions was the UK COVID-19 response strategy that continued throughout 2020, receiving criticisms from global health and epidemiology experts (e.g., Peto et al., 2020).

Early criticism drew attention to the UK's contact tracing approach, starting and then stopping against WHO advice, and the flaws in a national approach that failed to address regional variations and local outbreaks (Pollock et al., 2020). Extended periods of lockdown were said to increase social and economic damage, while periods of relaxation would trigger more deaths, with the most disadvantaged groups in society suffering the most in both scenarios (Peto et al., 2020). For instance, mental health across the UK had deteriorated by late April, with uneven effects across population segments, indicating persistence of socioeconomic and health inequalities (Pierce et al., 2020). A more regional approach and a system of tiers corresponding to perceived risk and level of restrictions was adopted later in 2020, before further national lockdowns were implemented in the winter, owing to new and more transmissible variants of the virus.

In the UK, gender reassignment and sexual orientation are protected characteristics under the Equality Act (UK Government, 2010) and require special consideration. Legal protection does not guarantee equality and inclusion in social/political/cultural life, however. The National LGBT Survey indicates inequalities among LGBTQIA+ populations compared to the general population (Government Equalities Office, 2018). For example, 70% of people still avoid being open about their

This article is protected by copyright. All rights reserved.

Accepted Article sexuality at work or in public for fear of negative reactions, and transgender people reported lower life satisfaction than cisgender people. By some measures, equality/inclusion is declining further; according to a recent survey, 65% of trans people in 2021 didn't reveal their gender identity at work compared to 52% in 2016 (Fisher and Jónsdóttir, 2021).

***The Brazil context***

The first case of COVID-19 in Brazil was recorded in February 2020, with the initial response involving patient (home) isolation and contact tracing (Rodriguez-Morales, 2020). Brazil went on to experience some of the highest COVID-19 infection rates and numbers of cases and deaths in the world, even with comparatively low testing rates (Marson, 2020). Despite past experience of public health emergencies, Brazil appeared ill-prepared, with early concerns about testing capabilities and the robustness of public health systems (Rodriguez-Morales, 2020). Concern was raised by national and international health science experts over Brazilians living in favelas, Indigenous populations (The Lancet, 2020), and potential health system collapse in inland municipalities (Marson, 2020). The first wave in April saw the Amazonian city of Manaus utilise mass graves to manage the increasing number of deaths (Phillips and Maisonnave, 2020).

Brazil's response to the crisis, led by ultra-conservative President Jair Bolsonaro, received national and international criticism. This included concerns over Brazil's response in general (e.g., social distancing was introduced but with limited public adherence) (Marson, 2020), and direct criticism of Bolsonaro (The Lancet, 2020). The President opposed WHO recommendations, minimised the illness and its impacts (publicly dismissing it as just a 'little cold' (McCoy and Traiano, 2020)), rejected scientific evidence (Oliveira et al., 2020), suppressed data, and politicised the health sector, whereby several government ministers either resigned or were dismissed over disagreements in Federal pandemic policies (Marson, 2020), leading to calls for Bolsonaro to "drastically change course or [he] must be the next to go" (The Lancet, 2020). Federalism and territorial power dynamics in Brazil resulted in "intergovernmental incoordination" (Abrucio et al., 2020) and asymmetrical approaches (Pereira et al., 2020) to the pandemic across different states, regions, and governments. Disagreements occurred between Mayors, Governors, and the President over crucial measures, such as the end of quarantine (supported by Bolsonaro and the Federal Government despite growing infection rates) (Marson, 2020). Such conflicts jeopardised the effectiveness of COVID-19 policy responses (Abrucio et al., 2020).

During COVID-19 in Brazil, many LGBTQIA+ people experienced job losses (#VOTELGBT, 2021), social and economic exclusion, fear of discrimination and violence (Cowper-Smith et al., 2021), and some insecure housing (Fisch, 2020). This reflects pre-existing inequalities and marginalisation, without adequate protections. While there have been some positive recent changes for LGBTQIA+ rights, e.g., legal self-determination for trans people in 2018, Brazil remains a hostile environment for LGBTQIA+ people owing to growing anti-gender and anti-gay rhetoric, exacerbated further by the election of Bolsonaro in 2018 (Cowper-Smith et al., 2021). Brazil has some of the highest rates globally of transphobia-related deaths (Wareham, 2020). President Bolsonaro himself is well-known for being homophobic, and a survey of LGBTQIA+ Brazilians revealed almost 99% of respondents were dissatisfied with his pandemic performance (VOTELGBT, 2021).

This article is protected by copyright. All rights reserved.

Accepted Article

### Methods

#### *Interviews*

Semi-structured interviews were conducted between May and September in the UK (conducted by the first author), and July and November in Brazil (conducted by the first and third authors), 2020, with people self-identifying as LGBTQIA+, over 18 years old and living in the UK or Brazil. This timeframe represents various stages of pandemic responses in the UK and Brazil, including periods of rising cases and deaths, lockdowns, and easing and tightening of restrictions – broadly the first wave of the global crisis. Interviews are highly valuable for the in-depth and contextualised insights they provide, delivering detailed understandings of individual lived experiences (Kusek and Smiley, 2014). Interviews were valuable for participants, with time and space to tell their stories, which was particularly significant during COVID-19 – interviews as enabling interactive engagement, catharsis, and feelings of empowerment (Hutchinson et al., 1994). All participants were questioned on the same topics, covering challenges faced, coping strategies, and improving crisis responses for LGBTQIA+ people. Interviews were undertaken in English or Portuguese, as per participant preference. Interviews were conducted via Zoom and audio-recorded, lasting on average 65 minutes each.

#### *Participants*

Participants were recruited through professional and personal networks (e.g., community organisations the authors are connected to), approaching relevant organisations identified online who work with LGBTQIA+ people in the UK (36 organisations) and Brazil (25 organisations), and posting information on social media pages/groups. Representatives of organisations and community groups, professional contacts, and managers of social media pages/groups promoted the study amongst their networks. Thus, snowball sampling was employed. Interested individuals emailed the researchers directly.

We completed 29 interviews: 17 for the UK and 12 for Brazil. Participants included a diverse range of LGBTQIA+ identities (Table 1).

*Table 1. Study participants with self-reported gender and sexuality*

| **United Kingdom** | | |
|---|---|---|
| **Participant** | **Gender** | **Sexuality** |
| Prince | Non-binary transmasculine person | Pansexual, queer, polyamorous |
| Justin | Non-binary transmasculine person | Non-definable |
| Lyndsey | Non-binary transgender woman | Queer |
| Amber | Transgender woman | Pansexual |
| Donna | Transgender woman | Lesbian |
| Emily | Transgender woman | Bisexual |
| Chad | Transgender man | Bisexual, polyamorous |
| Lloyd | Transgender man | Gay |
| Brittany | Cisgender woman | Bisexual, pansexual, queer |
| Carly | Cisgender woman | Pansexual |
| Ken | Cisgender man | Queer, gay |
| Kyle | Cisgender man | Gay |
| Raheem | Cisgender man | Gay |
| Sean | Cisgender man | Gay |

This article is protected by copyright. All rights reserved.

| | | |
|---|---|---|
| David | Cisgender man | Gay |
| Omar | Cisgender man | Gay |
| Lucas | Cisgender man | Gay |
| **Brazil** | | |
| **Participant** | **Gender** | **Sexuality** |
| Rafa | Non-binary, transfeminine person | Bisexual |
| Laura | Cisgender woman | Lesbian |
| Ursula | Cisgender woman | Lesbian |
| Tula | Cisgender woman | Lesbian |
| Jorge | Cisgender man | Bisexual |
| Edgar | Cisgender man | Gay |
| Arthur | Cisgender man | Gay |
| Bruno | Cisgender man | Gay |
| Heitor | Cisgender man | Gay |
| Jadir | Cisgender man | Gay |
| Pedro | Cisgender man | Gay |
| Mauro | Cisgender man | Gay |

***Recruitment and sample limitations***

Recruitment favoured people with internet access, which was required to respond to an online advert and use Zoom for the interview. This is especially salient when aiming to engage marginalised groups, who are not only potentially more vulnerable during crises but less likely to access internet technologies (Haworth et al., 2019).

The sample reinforces the notion that more visible voices within LGBTQIA+ populations can dominate LGBTQIA+ representations, especially for Brazil, where most participants were gay cisgender men. This prompts us to reflect on access more broadly and consider the inequalities *within* marginalised groups, if capacity for online participation is linked to relative economic advantage – what other services and benefits do more marginalised LGBTQIA+ people not have access to? However, we cannot be certain that internet access was the only factor influencing the Brazil sample, and other recruitment strategies may yield different results.

Other people and groups are under-represented. No participants identified as asexual or intersex. In the UK, only people from England and Scotland participated, meaning Wales and Northern Ireland are not included. All Brazil participants were from two states in the industrialised south-east of the country, Rio de Janeiro and São Paulo. The Brazil sample is not representative in terms of class, race and education, as all participants except two were white, and the majority were well-educated. Trans experiences are underrepresented in the Brazil sample. The accounts of the trans participant are analysed not as representative of all trans experiences, but to highlight some vulnerabilities associated with non-normative gender identities.

Such limitations do not reduce the value of the insights gained; this study emphasises individual lived experiences of LGBTQIA+ people and does not rely on representative samples. Additionally, some participants worked in LGBTQIA+ community support roles during COVID-19 and thus were able to inform of experiences of others across LGBTQIA+ populations. Even so, this study should be

Accepted Article

This article is protected by copyright. All rights reserved.

Accepted Article understood as presenting insights into some experiences of LGBTQIA+ people during the pandemic, rather than representing all experiences.

***Thematic analysis***
Interview recordings were transcribed verbatim for analysis by the first and second authors themselves to facilitate data immersion and familiarisation (Braun and Clarke, 2006). Identifying information was anonymised during transcription and names replaced with pseudonyms. Portuguese transcripts were translated to English to facilitate comparative analyses. Inductive coding was performed, and data analysis followed a combination of established thematic analysis approaches (Braun and Clarke, 2019); summarising the data under domains of interest (e.g., challenges faced or coping strategies), and seeking to identify deeper cross-cutting explanatory themes to describe patterns across the dataset in relation to LGBTQIA+ experiences.

***Positionality***
We believe it is important to make visible our positions and characteristics that are most relevant in shaping how we approached the research[5]. For this study we hold both insider and outsider positions (Kusek and Smiley, 2014). All three authors identify as part of the LGBTQIA+ population, and two are Brazilian. All three were living in the UK at the time of the interviews. In this way we share characteristics and experiences with many of our participants, including gender and sexuality, culture, and some experiences of marginality. But we are also outsiders in important ways. For example, we are not transgender and are relatively privileged socio-economically compared to several of our participants. Of significance for this study is that we experienced the COVID-19 pandemic at the same time as our participants. We too endured lockdowns, social-distancing and other restrictions, isolation, and anxiety, and benefited from the support of LGBTQIA+ peers.

Our personal connections to LGBTQIA+ people, organisations and spaces helped us gain access to information and participants. Although we do not claim to have personal understanding of everybody's experiences, our own exposures to, and experiences of, marginality as LGBTQIA+ people perhaps allowed us to listen more attentively and empathetically to our participants. We were also able to interpret people's stories through less dominant, cisheteronormative lenses. Concurring with Kusek and Smiley (2014), ignoring these positions would have been disadvantageous to the value of such personal research.

### Results and discussion

***Diversity within LGBTQIA+ experiences***
There is no universal LGBTQIA+ experience, but multiple histories and varied lives influenced by factors of gender, sexuality, class, age, race/ethnicity, religiosity, location, living conditions, and more. Interviews demonstrated vast diversity in LGBTQIA+ COVID-19 experiences. As examples, unique and unequal experiences were observed for LGBTQIA+ people of colour (POC), transgender people, and those with low or insecure incomes. These differences highlight the need to move beyond 'one size fits all' policy approaches.

Experiences of racialised identities
Racialised identity, and intersections with gender, sexuality, and class, was an important topic raised by participants in Brazil, sometimes even more important than being LGBTQIA+. As Laura describes, race and being Black was seen to negatively impact employment prospects and financial security,

This article is protected by copyright. All rights reserved.

Accepted Article and social networks – both key elements of vulnerabilities and coping capacities associated with the pandemic.

> "*We are going through a financial crisis that makes me feel unstable mentally. To think that we may not be able to have enough to eat at some point… it has been a challenge not to get fired… Of course, there is a gender issue, being a woman, but being a lesbian doesn't necessarily have as much impact, maybe because of the way I keep my personal and professional relationships separate… but racial issues are impactful. I've had many job interviews in my life, and I think it affects me much more than my sexual orientation, for example… The issue of loneliness of Black women is very serious, where our circle is not that large and complex as that of white people… During the pandemic, I learnt about Black lesbian and bisexual women groups being created for virtual meetings. They are aimed at discussing sexual and mental health, because some places have been closed during lockdown. The groups have also been organising crowdfunding to support low-income women*." – Laura, lesbian cisgender woman, Brazil.

This aligns with existing scholarship. Race and ethnicity have been significant contributors to increased vulnerability during many major crises (e.g., Hurricane Katrina; Laska and Morrow, 2006). While stress, suicide risk, and lived experiences of discrimination, stigma and fear are heightened among LGBTQIA+ populations compared to the general population, these are even higher for LGBTQIA+ POC (O'Donnell et al., 2011; Ghabrial, 2017), who are multiply marginalised (Cyrus, 2017), including through racism within LGBTQIA+ populations and homo/transphobia and heterosexism inside racial/ethnic groups, increasing their risk of mental and physical health problems (Ghabrial, 2017). A study by Ghabrial (2017) found that LGBTQIA+ POC felt disconnected from their racial/ethnic communities because of their sexual identity and disconnected from mainstream LGBTQIA+ communities because of their racial/ethnic minority identities. In this context, Ghabrial (2017) highlighted the value of queer spaces specifically for racialised identities. While Laura describes race as a cause of prejudice and discrimination, she also references a sense of belonging and solidarity through the Black women's groups. Singh and McKleroy (2011) previously showed a strong sense of pride in both racial/ethnic and gender identities was a key element of transgender POCs' resilience to traumas. Notably, race was mentioned in our study mainly by participants who identified themselves as Black or POC. That white participants could not identify with the lived experiences of LGBTQIA+ POC reinforces the need to consider the diversity of lives *within* minority groups and intersecting axes of marginalisation for more inclusive crisis planning.

***Transgender experiences***

Significantly, transgender lives differ to cisgender lives, influencing pandemic experiences in several important ways. While cisgender people experienced disruptions to healthcare access, this issue was particularly significant for transgender patients (see also López, 2021). Access to affordable, trans-positive healthcare has been reported as a critical aspect of resilience to trauma for transgender people, especially trans POC (Singh and McKleroy, 2011). For many individuals, healthcare relating to gender affirmation and gender identities was delayed if not halted completely during COVID-19, often without communication, as Prince describes below. This caused confusion and anxiety, exacerbated mental health concerns, and added to already long waiting periods. These delays, coupled with people being at home with their bodies more and reduced access to support, also intensified gender dysphoria.

This article is protected by copyright. All rights reserved.

Accepted Article

"*A lot of my health things have been paused… I was meant to, if COVID hadn't happened, have top surgery this summer. And that's clearly been pushed back, but I haven't been sent a letter or phone call to be told that… It's particularly heart-breaking. I had a personal goal that a lot of my medical transition would've been done by the time I was 30. And considering that I began publicly coming out as trans when I was like 22, I didn't think that would've been such a difficult hurdle, but it's turned out to be like that.*" – Prince, pansexual polyamorous, queer and non-binary transmasculine person, UK.

Some transgender participants described a 'double anxiety' of being in public; one associated with the pandemic and another around transphobic abuse they had experienced.

"G*oing outside has been quite tricky because I've got two anxieties stacked on top of each other. One is whether people are going to stay 2 meters apart, whether other people are going to obey the rules. And the second one is whether people are going to be staring at me in the street because I'm so early in my transition that I still look obviously trans and I don't pass at all."* – Donna, lesbian transgender woman, UK.

Here we relate theories of racism and racialised bodies to transphobia and scrutiny of trans bodies. Bolin and Kurtz (2018) describe how a white immigrant can stop being 'ethnic' in ways that a POC cannot avoid being labelled Black (or brown or Arab or Latino) and 'other' with accompanying marginalising effects by dominant discourses. Arthur relayed the same sentiment in our study, referencing the relative safety he experiences through being read as white and heterosexual.

*"I am not an effeminate person, which protects me a lot, compared to what other effeminate people go through, and I am not black either, although my father is black and my mother is white – I do not consider myself white, but society does, which also protects me from a lot of things. As a LGBT person, people only know that I am gay when I mention it."* – Arthur, gay cisgender man, Brazil.

Similarly, (visibly) trans people cannot stop being trans in public in the way that cisgender LGB people may be able to conceal their sexuality in mainstream society where necessary to remain safe (see *stigma concealment;* Ghabrial, 2019). This difference represents potentially increased discrimination for transgender people, including public verbal or physical abuse and other consequences like reduced stable employment opportunities compared to cisgender people, constituting a further inequality *within* LGBTIQ+ populations. Ghabrial (2019) explores the complexities of queer identity visibility further, through study of bisexual women and gender diverse POC, describing how while the act of "passing" can allow respite from social stigma and access to resources and safety, the invisibility of identities can also negatively impact the self-worth, wellbeing and access to support for those who "pass" in one way or another (e.g., a bisexual woman who is read as heterosexual through her relationship with a man may be denied access to aspects of queer community resilience).

***Employment and financial inequalities***

Inequalities also shaped pandemic experiences in financial terms. LGBTQIA+ people are more likely to work in affected industries, and unemployment and salary reductions during COVID placed pressures on many livelihoods with a range of consequences (Banerjee and Nair, 2020).

"*That is the biggest issue, in my view – impact on income, resulting in some people having to go back to a home that is not always welcoming.*" – Ursula, lesbian cisgender woman, Brazil.

This article is protected by copyright. All rights reserved.

Accepted Article

But pressures were not felt uniformly, as Ken highlights below. Inequality within LGBTQIA+ populations meant that while some people struggled to survive on minimal income, those with more secure employment and wealth were less pressured financially and not exposed to other potential challenges, such as having to change living arrangements. Some more economically privileged people even found lockdowns beneficial, for example increasing their productivity through working at home or having extra time for learning new skills, perhaps widening existing inequalities.

> "*I'm pretty privileged but there's large parts of our community who are dependent on the night time economy for example… and I've seen all of their work completely disappear and they've gone from being active parts of the community and earning money to earning nothing and suddenly finding out about the realisation of universal credit… having like 150 quid a month to live on… I've seen people moving in together to save costs… particularly younger trans or non-binary people seem disproportionately hit, and particularly kind of female, bi and lesbian women, and disabled people… in comparison to my other white cis gay male friends. They're all working from home doing coding or whatever and taking time to paint their dining room and things like that. Whereas other parts of the community are struggling.*" - Ken, gay cisgender man, UK.

Participants in both the UK and Brazil showed awareness of such complexities and inequalities and expressed concern about the lack of protection for the vast majority of LGBTQIA+ people, and trans people in particular as victims of exclusion and violence. Particularly in Brazil, the people interviewed generally described themselves as socio-economically privileged and highlighted that their experiences were not representative of the entire LGBTQIA+ population, with greater disadvantage and vulnerability befalling others, especially trans people and those with low-income and who are precariously employed, including sex workers. We posit that such awareness of LGBTQIA+ lives, diversity and inequalities is lower among the general non-LGBTQIA+ population, and thus advocate for the inclusion of (a variety of) LGBTQIA+ people themselves in designing and implementing crisis responses and broader public policies.

***Common experiences despite varying contexts***

Alongside diverse experiences within LGBTQIA+ populations, and differences across the varying contexts of Brazil and the UK, some experiences appeared almost universal in our data. While more nuanced analyses of specific topics are necessary, and these will occur in subsequent outputs, here we aim to showcase commonalities in LGBTQIA+ experiences of the crisis across national, governmental, social, and cultural boundaries, allowing us to draw attention to underlying processes of marginalisation that pervade as root causes of vulnerabilities in varying contexts.

***Key challenges: mental health, isolation, and discrimination***

While many people experienced mental health challenges during the pandemic, including anxiety and low mood, Gorman-Murray et al. (2017, p. 44) inform that these issues "have a more serious impact on marginalised groups who often already evidence higher fear, stress, anxiety, and depression in day-to-day life due to their more limited access to social capital and political inclusion, and who are then further side-lined in disasters". Mental health concerns, especially in lockdown, including isolation, depression and anxieties were widespread across both UK and Brazil interviewees[6]. People experienced new anxieties as well as exacerbation of past issues, both related to the virus itself and specific issues related to gender or sexual identity. People often didn't know where to turn for support, especially with many LGBTQIA+ services disrupted or overwhelmed by

This article is protected by copyright. All rights reserved.

increased demand during the pandemic (LGBT Foundation, 2020). Prince relayed an experience of calling a general population mental health helpline in the UK only to be left disillusioned by the experience with the helpline agent unable to understand issues specific to trans lives.

Isolation from support networks and identity-affirming spaces caused significant heightened stress. Some participants described being at home with unsupportive families, while others reported having limited biological family relationships or being cut off from friendship circles, reducing the support networks that so many people relied on in the pandemic.

> "*People in the LGBT community are often isolated from their families. They may have lost friends in coming out. They've often moved into this area because they think it's going to be a happier place to live. So, once you say to them 'stay home, don't go to LGBT spaces - to your community groups', they're finding themselves very isolated, and very lonely and depressed… It's not been good for me; it's not been good for my mental health… I came here seeking to be part of a community and then all of a sudden was shut off completely."* – Lloyd, gay transgender man, UK.

The LGBT Foundation (2020) reports older adults are a group particularly vulnerable to isolation, especially those living alone like Lloyd. As older people were the most at-risk demographic to the virus, they were more likely to be shielding and less able to return to public and social life when restrictions were loosened, compared to younger people. Additionally, if origin families are limited, 'chosen families' are likely to be people of a similar age, meaning for older people their friends may have also been staying at home more for their safety, further adding to isolation.

More widely, disruptions to (physical) LGBTQIA+ spaces were acutely felt, including nightlife and queer parties, but also community spaces, support groups and activities like Pride festivals. In 2020, hundreds of Pride festivals were postponed or cancelled globally, reducing chances for LGBTQIA+ communities to come together, socialise, share concerns, and celebrate social change achievements (digital events were valuable, but cannot replace personal gatherings) (Banerjee and Nair, 2020). LGBTQIA+ spaces usually provide many people with essential opportunities to express themselves and their identities freely and safely.

> "*I've found it really disruptive not having clubs to go to to release the tension of existing in a very cisgendered heterosexual society and needing to escape... So, on top of losing work, I also lost a place to truly express myself."* – Lyndsey, queer transgender woman, UK.

Outside of these spaces, the potential for LGBTQIA+ people to experience discrimination and stigma increases, including greater risk of exposure to violence. Mauro describes fear associated with being gay in Brazil and limited social circles being exacerbated by the pandemic.

> "*In Brazil, there are some things happening that I am terrified of... discourses of hatred and violence... I think everyone is feeling fragile during the pandemic. The fact that I am LGBT... what is going to happen to us... but above all, what I've been mostly impacted upon is the reduced social network due to the stigma of being LGBT.*" – Mauro, gay cisgender man, Brazil.

While Amber describes changing her shopping habits to protect her safety.

> "*I used to shop in [a major supermarket], and I'd had a bit of crap by people in the store, following me around and name-calling and the security guards not being bothered and stuff*

This article is protected by copyright. All rights reserved.

Accepted Article

Accepted Article

*like that. So that's why I switched my shopping habit [to online]."* – Amber, pansexual transgender woman, UK.

Participants in the UK and Brazil described a perception of increased LGBTQIA+ discrimination during the pandemic, including verbal abuse in public, but especially online on social media, and particularly directed towards transgender and non-binary identities.

"*It's probably quite telling that transphobia has spiked in this time, that we're seeing so much more of it in the media and normalised. And it could also be a case that people are scared and they feel like they're more in control if they can shout at someone… I'm not on Twitter on purpose… I've noticed more transphobia online*."- Prince, pansexual polyamorous, queer and non-binary transmasculine person, UK.

Digital and social media are important to LGBTQIA+ people in everyday life as safe(r) spaces to discover more about their gender and sexuality, seek potential partners, form friendships, and participate in queer activism, sometimes anonymously and often more openly than in offline environments (Pickles, 2020). By contrast, these same environments provide opportunities for hate speech and discrimination against LGBTQIA+ people, to which transgender people are disproportionately prone (Pickles, 2020; Ștefăniță and Buf, 2021). Exposure can cause mood swings, anger, loneliness and fear in the short term, and erosion of social trust and negative effects on personal development in the longer term (Ștefăniță and Buf, 2021). That increased transphobia and discrimination online has been perceived at a time when people are both more isolated and anxious and likely spending more time online due to COVID restrictions is especially troubling. Social media was a useful platform during the pandemic for many people, including LGBTQIA+ people (Fisch et al., 2020), helping them connect with friends and family, find information, and be distracted and supported during isolation at home. However, access to the benefits of these spaces and resources was not shared equally, with online discrimination being a source of emotional distress for some.

Pedro was one of several participants who felt that the pandemic exacerbated discrimination against LGBTQIA+ people through HIV-related stigma.

"*In Brazil, this was much emphasized: it [COVID-19] will only affect the LGBTQ+ group, because it will be like HIV. And these are two completely different things and I find it very offensive*." – Pedro, gay cisgender man, Brazil.

Stigmatisation, a social process that excludes those perceived to spread disease or threaten mainstream public life, during COVID-19 has impacted various societal groups (Bhanot et al., 2020). As with historical crises like HIV, Hurricane Katrina or the Ebola outbreak, LGBTQIA+ people were blamed in numerous countries for COVID-19, leading to greater stigma, discrimination, and violence (Bishop, 2020). Stigma has a range of individual and public health consequences pertinent to and on top of challenges already faced during the pandemic, such as social isolation, deteriorated psychological health, and deterrence from reporting symptoms, getting tested and seeking treatment, impeding early detection and control of the virus (Bhanot et al., 2020).

***Coping strategies and the importance of community groups***

Participants drew on coping strategies developed through past experiences of distress to help them during the pandemic. They described adopting regular daily routines for activities like work, exercise and sleep, monitoring diet and alcohol consumption, and talking about their challenges and feelings to friends or therapists. Neither UK nor Brazil government responses provided any LGBTQIA+-specific

This article is protected by copyright. All rights reserved.

guidance. In Brazil, people struggled to find information on how to manage their wellbeing and stay safe generally, with each level of government (City, State, and Federal) communicating different and contradictory guidelines, fostering misinformation and heightened stress. Without clear instructions, individuals determined their own protective measures, including staying and working at home where possible, physical distancing, limiting social circles, prioritising certain activities or self-quarantining before meeting vulnerable people, and following information from outside sources like WHO. Decisions were often based on personal perceptions of relative risk, as Jorge described regarding contracting/spreading the virus versus mental wellbeing.

> "*It's a conscious risk to visit my in-laws [in the same neighbourhood]; but then it came to a point everyone was thinking about it... after five months in lockdown, we needed some level of mental health.*" – Jorge, gay cisgender man, Brazil.

Even when physically distant, LGBTQIA+ people created opportunities for socialising and reducing loneliness. Social media, video calls, dating apps and other online activities became more frequent for connecting with existing and new contacts, often also LGBTQIA+ people, and for exchanging support with friends, community groups and services. Online social platforms have been associated with social capital as an important element of resilience during crises, facilitating the perseveration and strengthening of existing social relations and the creation of new connections (Haworth et al., 2018). However, inequalities within LGBTQIA+ populations meant that these privileges were not available to everyone.

Vital to coping with the pandemic were LGBTQIA+ organisations and community and peer-support groups. Existing and new community networks provided supportive information (ANTRA, 2021), mutual aid, and safe and identity-affirming (online) spaces to share experiences and connect with others. In the absence of family or more formal support, community and charity groups delivered mental health support and helped reduce social isolation. Moving activities online also presented opportunities to connect with new and diverse audiences, in some ways improving accessibility to activities. Those in organising roles reported that running community activities gave them a sense of purpose and life-motivation during the pandemic.

> "*I work for an LGBTIQ+ organisation… We provide welfare for LGBTQI+ Muslims… With COVID we've completely had to change the way we support our members… We started doing stuff online, and it's been amazing because we started reaching out to bigger communities and people further afield… we had a Pride event and we reached out to countries like Saudi Arabia, Egypt, where being homosexual is against the law and you would be imprisoned or even worse, sentenced to death. So, we had people sitting in their bedroom saying 'wait a minute, I'm in Saudi Arabia. It's 3am, I'm listening to your event and I'm actually at a Pride!' So, our reach was huge! It was amazing.*" – Omar, gay cisgender man, UK.

Others have recorded similar findings. Cowper-Smith, Su and Valiquette (2021) noted the lack of LGBTQIA+ inclusion in Brazil COVID-19 responses and highlighted the importance of community groups and grassroots organisations in filling gaps, in particular for providing safe shelters for LGBTQIA+ asylum seekers. This is part of the broader pattern of LGBTQIA+ people in Brazil being left to support themselves during the pandemic, with another example being the LGBTQIA+ shelter in Rio de Janeiro, Casa Nem, enforcing their own lockdowns and protective measures to keep vulnerable people safe (Fisch, 2020), and distributing basic food-parcels (Barreto, 2020). Similarly, reports in the UK describe how, despite reporting reduced income and resources during COVID, LGBTQIA+

This article is protected by copyright. All rights reserved.

Accepted Article

Accepted Article community and mutual-aid organisations have played various important roles in the pandemic, from organising social activities to reduce isolation to delivering food, medication, and safer sex resources (LGBT Foundation, 2020). These resilience, coping, and mutual aid capacities warrant further scholarly attention and should be considered when planning for crises and allocating funding and resources, with a view towards future risk reduction rather than crisis response.

***Cisheteronormativity and LGBTQIA+ vulnerabilities***

Experiences of LGBTQIA+ people in both the UK and Brazil are linked to pre-existing socio-political marginalisation and economic inequality across societies. For example, it was already known in the UK that LGBTQIA+ people (especially trans people) were more likely to suffer mental health challenges without adequate support, and that LGBTQIA+-friendly spaces, including nightlife and Pride events, are highly important but in decline (Government Equalities Office, 2018) – indicating causes that pre-date the pandemic. Likewise in Brazil, LGBTQIA+ populations already experienced poor mental health pre-pandemic (Oliveira et al., 2020), and despite the lack of official data, academic and social movement reports identify high levels of unemployment and sub-employment amongst trans people (Ribeiro et al., 2021). Yet the intensification of these issues during COVID-19 was not adequately planned for in crisis policies and responses.

These experiences born from marginality remind us that we must look beyond identifying who is vulnerable in moments of crisis and short-term services addressing some needs, and instead examine the root causes and how dominant structures and approaches in society work to exclude and marginalise in 'normal' times. We found cases in both the UK and Brazil of transgender people perceiving lockdowns and social distancing as positive experiences, providing a temporary escape from 'normal' life. This included: enabling some people to transition medically or personally in private at home away from social pressures; and more broadly as reprieve from the pressures of being trans in everyday life, such as transphobic discrimination, violence, and public scrutiny of trans bodies.

The key roles played by LGBTQIA+ community groups in supporting coping capacities during the pandemic echo pre-pandemic needs and activities. Such groups developed in 'normal' times due to existing gaps in services and exclusion from mainstream society, with LGBTQIA+ people developing their own supportive and safe spaces. What we saw in the pandemic was in principle largely the same groups doing what they have always done to support each other in the absence of adequate LGBTQIA+ inclusion in society and public policies, but perhaps in some new or different ways, rather than new challenges and community responses arising.

These statements on pre-existing marginalisation and vulnerabilities are nothing that disaster vulnerability theory doesn't already tell us (e.g., Wisner et al., 2001). But then why are LGBTQIA+ needs still largely absent from crisis policies, given we should be able to predict these existing issues will be exacerbated? Even international frameworks aimed squarely at building resilience capacities for the most socio-economically vulnerable groups in societies fail to adequately include LGBTQIA+ populations, such as the Sendai Framework for Disaster Risk Reduction or the Sustainable Development Goals. A key factor limiting LGBTQIA+ inclusion is dominant cisgender and heteronormative assumptions pervading in disaster policy development and crisis planning and responses (Dominey-Howes et al., 2014).

Social vulnerability approaches often problematically assign the label of 'vulnerable' to communities based on demographics (e.g., based on class, race, gender, etc), which then serve as measures of

This article is protected by copyright. All rights reserved.

Accepted Article vulnerability, without incorporating community knowledge of how vulnerability looks or feels to those who experience it, or critical assessment of the oppressive structures and processes that create marginality (Jacobs, 2019). If marginality is not inherent to individuals but produced through societal and political processes that increase disadvantage and exclusion, then the structures and conditions of privilege and the role of those advantaged by power in dominant oppressive structures must also be critiqued (Pease, 2010). For activism, change, and inclusion for minorities in crisis planning and public policies, oppressive structures need to be named as such (Jacobs, 2019), enabling movement past labelling gender and sexuality (and race, ability, class…) as vulnerability indicators, and towards addressing structural factors of cisheteronormativity and heterosexism (and racism, ableism, classism…).

**Recommendations**

***Improving policies for LGBTQIA+ people***

In the short term, policies should prioritise access to disrupted services that were directly related to the aspects of LGBTQIA+ vulnerability most exacerbated during COVID-19 lockdowns. This should include facilitating increased and targeted mental health support, maintaining access to healthcare during crises, especially transgender care, and providing financial and housing aid to those most economically-disadvantaged, particularly in Brazil. Resources should be allocated to support and expand upon the important work already being done by LGBTQIA+ community, volunteer, and mutual aid groups. This could include appointing LGBTQIA+ liaison personnel within COVID-19 recovery groups and linking government and non-government activities through strategic partnerships to facilitate knowledge sharing. The scope, reach, and public awareness of LGBTQIA+ public services, such as LGBTQIA+ reference and citizenship centres across Brazil, must also be expanded, and their staff protected from threats – including from COVID-19, but also temporary and unstable contracts and funding (Cassal, 2018).

For longer-term change, policies cannot continue being based on assumptions that populations are cisgender and heterosexual. They must not only include and account for LGBTQIA+ needs and capacities but must recognise diversity *within* LGBTQIA+ populations. To better incorporate LGBTQIA+ diversity, education on the lives of gender and sexual minorities should be included as part of workplace training programmes for government and crisis management organisations, and LGBTQIA+ councils and advisory boards should be strengthened. Co-production of policies should be a goal; involving communities and harnessing community wisdom. Productive dialogue with LGBTQIA+ people themselves and the groups and organisations that represent them is essential for developing inclusive and efficient strategies. As, like Jadir says in the title of this paper, LGBTQIA+ people themselves have the best knowledge and understanding about LGBTQIA+ lives to inform what is needed for appropriate care and support.

Developing an international standard on LGBTQIA+ inclusive crisis responses and risk reduction will aid in preventing exacerbation of existing and creation of new inequalities during crises. As we have shown, while LGBTQIA+ populations are diverse and individual lives vary across geographic contexts, there are commonalities in vulnerabilities and coping strategies. An international standard is necessary to address common, structural, and cross-national causes of marginalisation and uneven crisis experiences.

For further description of UK and Brazil *policy recommendations* arising from this study see Haworth, 2021 and Cassal et al., 2021.

This article is protected by copyright. All rights reserved.

Accepted Article

**Further research**

This research focused on the first wave of the pandemic in 2020, but longer term or reflective studies traversing the changing stages of the pandemic and associated responses across the world will likely expound different and further unique challenges and coping strategies of LGBTQIA+ people. This might include examination of experiences of later waves of the virus and further public restrictions, but also longer-term recovery from the crisis and potential lingering impacts in the coming years. For example, how do LGBTQIA+ communities rebuild their social lives after extended periods of isolation? How can community and public LGBTQIA+ centres and shelters continue to offer services safely in the middle of a pandemic? What are the longer-term implications for trans healthcare systems and affected individuals of disruptions to trans medical transition services and gender affirmation treatments? Or what are the effects of high numbers of deaths, erasing LGBTQIA+ stories, memories, work, and experiences?

Beyond COVID, further research is needed on LGBTQIA+ experiences of crises generally, with larger and more diverse participant samples, particularly focusing on less-visible minorities *within* LGBTQIA+, such as non-binary, intersex, or asexual people. To comprehend the marginality and resilience of gender and sexual minorities more fully, studies must more acutely examine the intersections of gender and sexuality with other identity characteristics and axes of oppression, such as race and class. And researchers must not only investigate experiences of those deemed 'vulnerable', but critically examine the root causes of vulnerabilities and the structures of privilege that inform crisis management, aiming to address barriers to greater equality, diversity, and inclusion for minority groups in government and other organisations, social and health services, and crisis policies.

**Conclusions**

This study adds to the growing literature seeking to understand the experiences of gender and sexual minorities during crises, highlighting diversity that must be considered in future public health planning. Interviews in the UK and Brazil during the first wave of COVID-19 revealed unique vulnerabilities experienced by LGBTQIA+ people, as well as resilience and coping capacities that can be better harnessed and supported, ultimately to develop and implement more nuanced and inclusive social, health, and humanitarian policies and strategies. Common across our study participants' experiences was the influence of pre-existing economic inequality and social marginalisation, and 'one size fits all' crisis approaches that fail to recognise the heterogeneity of diverse populations.

Moving forward, researchers, policymakers and practitioners must shift focus away from crisis response towards risk reduction. If we were more prepared and marginalised sectors of society were better supported, we would see less severe impacts, as disaster studies have informed (Kelman, 2020). Crisis policies must not only account for LGBTQIA+ needs and capacities but must recognise diversity *within* LGBTQIA+ populations and intersections with other identity characteristics and axes of oppression. We must work now to address the political, economic, and social structures that constitute root causes of vulnerabilities to mitigate against future hazards, pandemic or otherwise. LGBTQIA+-inclusion in crisis planning and response requires longer-term attention beyond the immediate challenges of COVID-19 and a radical redefining of the status quo through working to recognise and resist cisheteronormative systems that create and reproduce inequalities.

This article is protected by copyright. All rights reserved.

**Recommended citation**
Haworth, B.T., L.C.B. Cassal, and T. de P. Muniz (2022) "No-one knows how to care for LGBT community like LGBT do": LGBTQIA+ experiences of COVID-19 in the UK and Brazil. *Disasters.*

**Correspondence**
**Billy Tusker Haworth** School of Geosciences, University of Sydney, Australia, and Humanitarian and Conflict Response Institute, University of Manchester, United Kingdom. Email: billy.haworth@sydney.edu.au

**Acknowledgements**
We thank the study participants, Dr Stephanie Rinaldi, and Dr Paola Eiras.

**Funding**
This project was supported by the Economic and Social Research Council, UK.

**Data Availability Statement**
Research data are not shared.

## References

Abrucio, F.L, E.J. Grin, C. Franzese, C.I. Segatto, and C.G. Couto (2020) 'Combating COVID-19 under Bolsonaro's federalism: a case of intergovernmental incoordination.' *Revista de Administração Pública*. 54(4). pp. 663-677.

ANTRA (Associação Nacional de Travestis e Transexuais) (2021) 'Cartilhas e manuais.' https://antrabrasil.org/cartilhas/ (last accessed on 10 August 2021).

Anzaldúa, G. (1991) 'To(o) Queer the Writer—Loca, escritora y chicana'. *Versions: Writing by Dykes, Queers and Lesbians*. Press Gang, Vancouver. pp. 249-263.

Banerjee, D. and V. Nair (2020) '"The untold side of COVID-19": Struggle and perspectives of the sexual minorities'. *Journal of Psychosexual Health*. 2(2). pp. 113-120.

Barreto, B.S (2020) 'Subcidadania LGBTQ e proteção social na pandemia de COVID-19'. *Revista do NESEF.* 9(2). pp. 32-50

Bhanot, D., T. Singh, S. Verma, and S. Sharad (2020) 'Stigma and discrimination during COVID-19 pandemic'. *Frontiers in public health*. 8. Article No. 829. https://doi.org/10.3389/fpubh.2020.577018 (last accessed on 10 August 2021).

Bishop, A. (2020) 'Vulnerability Amplified: The Impact of the COVID-19 pandemic on LGBTIQ People'. *Outright International*. https://outrightinternational.org/content/vulnerability-amplified-impact-covid-19-pandemic-lgbtiq-people (last accessed on 10 August 2021).

Bolin, B. and L.C. Kurtz (2018) 'Race, class, ethnicity, and disaster vulnerability'. In H. Rodríguez, W. Donner and J.E. Trainor (eds) *Handbook of Disaster Research.* Springer, Cham. pp. 181-203.

Braun, V. and V. Clarke (2006) 'Using thematic analysis in psychology'. *Qualitative Research in Psychology*. 3(2). pp. 77-101.

Braun, V and V. Clarke (2019) 'Reflecting on reflexive thematic analysis'. *Qualitative Research in Sport, Exercise and Health.* 11(4). pp. 589-597.

This article is protected by copyright. All rights reserved.

Accepted Article

Accepted Article

Cassal, L.C.B. (2018) 'Um estudo entre ruíunas: o programa Rio Sem Homofobia e a política LGBTI fluminense'. *REBEH – Revista Brasileira de Estudos da Homocultura*. 1(4). pp. 124-162.

Cassal, L.C.B., B.T. Haworth and T. de P. Muniz (2021) 'Aprendendo com experiências de pessoas LGBTI+ durante a Covid-19 em SP e RJ'. *HCRI Policy Brief Series*. https://www.hcri.manchester.ac.uk/research/publications/policy-brief-series/lgbtiq-covid-experiences/lgbtiq-covid-experiences-brazil/ (last accessed on 26 October 2022)

Chin-Hong, P., K. Alexander, N. Haynes, M. Albert and The Association of Black Cardiologists (2020) 'Pulling at the heart: COVID-19, race/ethnicity and ongoing disparities'. *Nature Reviews Cardiology.* 17. pp. 533-535.

Cowper-Smith, Y., Y. Su and T. Valiquette (2021) 'Masks are for sissies: the story of LGBTQI+ asylum seekers in Brazil during COVID-19'. *Journal of Gender Studies*. https://doi.org/10.1080/09589236.2021.1949970 (last accessed on 10 August 2021).

Crenshaw, K. (1991) 'Mapping the margins: Intersectionality, identity politics, and violence against women of color'. *Stanford Law Review*. 43(6). pp. 1241-1300.

Cyrus, K. (2017) 'Multiple minorities as multiply marginalized: Applying the minority stress theory to LGBTQ people of color'. *Journal of Gay and Lesbian Mental Health.* 21(3). pp. 194-202.

Dominey-Howes, D., A. Gorman-Murray and S. McKinnon (2014) 'Queering disasters: on the need to account for LGBTI experiences in natural disaster contexts'. *Gender, Place and Culture*. 21(7). pp. 905-918.

Dominey-Howes, D., A. Gorman-Murray and S. McKinnon (2016) 'Emergency management response and recovery plans in relation to sexual and gender minorities in New South Wales, Australia'. *International Journal of Disaster Risk Reduction*. 16. pp. 1-11.

Fisch, Y. (2020) 'Brazil LGBTQ group hides from virus in Copacabana building'. *AP News*. July 10. https://apnews.com/article/b9f984f0c15c7250733d3c6dcd7a69b0 (last accessed on 10 August 2021).

Fisher, F. and U.S.K. Jónsdóttir (2021) *Trans employee experiences survey: Understanding the trans community in the workplace*. Totaljobs. https://www.totaljobs.com/advice/trans-employee-experiences-survey-2021-research-conducted-by-totaljobs (last accessed on 14 March 2022).

Ghabrial, M.A. (2017) "Trying to figure out where we belong": Narratives of racialized sexual minorities on community, identity, discrimination, and health. *Sexuality Research and Social Policy.* 14(1). pp. 42-55.

Ghabrial, M.A. (2019) "We can shapeshift and build bridges": Bisexual women and gender diverse people of color on invisibility and embracing the borderlands. *Journal of Bisexuality*, 19(2). pp. 169-197.

Gorman-Murray, A., S. McKinnon, D. Dominey-Howes, C. Nash and R. Bolton (2018) 'Listening and learning: giving voice to trans experiences of disasters'. *Gender, Place and Culture*. 25(2). pp. 166-187.

Gorman-Murray, A., S. Morris, J. Keppel, S. McKinnon and D. Dominey-Howes (2017) 'Problems and possibilities on the margins: LGBT experiences in the 2011 Queensland floods'. *Gender, Place and Culture*. 24(1). pp. 37-51.

This article is protected by copyright. All rights reserved.

Accepted Article

Government Equalities Office, UK (2018) *National LGBT survey: Research report*. https://www.gov.uk/government/publications/national-lgbt-survey-summary-report (last accessed on 10 August 2021).

Haworth, B.T. (2021) 'Learning from LGBTIQ+ experiences of COVID-19 in the UK for future crises'. *HCRI Policy Brief Series*. pp. 1–21.

Haworth, B.T. (2022) 'The importance of queer community resilience'. *Australian Journal of Emergency Management*. 37(1). pp. 31–32.

Haworth, B.T, E. Bruce, J. Whittaker and R. Read (2018) 'The good, the bad, and the uncertain: Contributions of volunteered geographic information to community disaster resilience'. *Frontiers in Earth Science.* 6. Article No. 183. https://doi.org/10.3389/feart.2018.00183 (last accessed on 10 August 2021).

Haworth, B.T., C. Eriksen, and S. McKinnon (2019) 'Online tools can help people in disasters, but do they represent everyone?' *The Conversation.* May 29. https://theconversation.com/online-tools-can-help-people-in-disasters-but-do-they-represent-everyone-116810 (last accessed on 10 August 2021).

hooks, b. (1981) *Ain't I a Woman: Black Women and Feminism*. Pluto Press, London.

Hopkins, P. (2019) 'Social geography I: Intersectionality'. *Progress in Human Geography*. 43(5). pp. 937-947.

Hutchinson, S., M. Wilson, and H. Wilson (1994) 'Benefits of participating in research interviews'. *Journal of Nursing Scholarship*. 26(2). pp. 161-164.

Jacobs, F. (2019) 'Black feminism and radical planning: New directions for disaster planning research'. *Planning Theory*. 18(1). pp. 24-39.

Kelman, I. (2020) *Disaster by Choice*. Oxford University Press, Oxford and New York.

Kluge, H.H.P., Z. Jakab, J. Bartovic, V. D'Anna, and S. Severoni (2020) 'Refugee and migrant health in the COVID-19 response'. *The Lancet.* 395(10232). pp. 1237-1239.

Kusek, W. and S. Smiley (2014) 'Navigating the city: gender and positionality in cultural geography research'. *Journal of Cultural Geography*. 31(2). pp. 152-165.

Lancet, The. (2020) 'COVID-19 in Brazil: "So what?"'. *Lancet*. 395(10235). pp. 1461.

Laska, S. and B.H. Morrow (2006) 'Social vulnerabilities and Hurricane Katrina: an unnatural disaster in New Orleans'. *Marine technology society journal*. 40(4). pp. 16-26.

Lee, H.S. and V. Miller (2020) 'The disproportionate impact of COVID-19 on minority groups: a social justice concern'. *Journal of Gerontological Social Work.* 63(6-7). pp. 580-584.

LGBT Foundation (2020) *Hidden Figures: the impact of the COVID-19 pandemic on LGBT communities in the UK – 3rd edition.* https://lgbt.foundation/coronavirus/hiddenfigures (last accessed on 10 August 2021).

López, J. (2021) 'The Covid-19 pandemic and its impact on transition related healthcare in the UK'. Research Report, University of Bradford. https://static1.squarespace.com/static/5e8a0a6bb02c73725b24dc9d/t/60fd4c3971a540627227eb

This article is protected by copyright. All rights reserved.

4e/1627212859946/Trans+Health+and+Covid+Summary+Report+2021.pdf (last accessed on 10 May 2022).

McKinnon, S., A. Gorman-Murray, and D. Dominey-Howes (2017) ‘Disasters, queer narratives, and the news: how are LGBTI disaster experiences reported by the mainstream and LGBTI media?’. *Journal of homosexuality*. 64(1). pp. 122-144.

Madrigal-Borloz, V. (2020) ‘Violence and discrimination based on sexual orientation and gender identity during the coronavirus disease (COVID-19) pandemic’. *United Nations*. https://undocs.org/A/75/258 (last accessed on 10 August 2021).

Marson, F.A.L. (2020) ‘COVID-19 – 6 million cases worldwide and an overview of the diagnosis in Brazil: a tragedy to be announced’. *Diagnostic Microbiology and Infectious Disease.* 98(2). Article No. 115113. https://doi.org/10.1016/j.diagmicrobio.2020.115113 (last accessed on 10 August 2021).

McCoy, T. and H. Traiano (2020) ‘Brazil’s Bolsonaro, channeling Trump, dismisses coronavirus measures - it’s just ’a little cold’’. *The Washington Post*. March 25. https://www.washingtonpost.com/world/the_americas/brazils-bolsonaro-channeling-trump-dismisses-coronavirus-measures--its-just-a-little-cold/2020/03/25/65bc90d6-6e99-11ea-a156-0048b62cdb51_story.html (last accessed on 10 August 2021).

O'Donnell, S., I.H. Meyer, and S. Schwartz (2011) ‘Increased risk of suicide attempts among Black and Latino lesbians, gay men, and bisexuals’. *American journal of public health.* 101(6). pp. 1055-1059.

OHCHR (Office of the United Nations High Commissioner on Human Rights) (2020) ‘COVID-19: the suffering and resilience of LGBT persons must be visible and inform the actions of States’. *United Nations.* https://www.coe.int/en/web/commissioner/-/covid-19-the-suffering-and-resilience-of-lgbt-persons-must-be-visible-and-inform-the-actions-of-states (last accessed on 10 May 2022).

Oliveira, F., H. Carvalho, and J.G. Jesus (2020) ‘LGBTI+ em tempos de Pandemia de Covid-19’. *Diversitates International Journal*. 12(11). pp. 60-94.

Pease, B. (2010) *Undoing privilege: Unearned advantage in a divided world.* Zed Books Ltd, London and New York.

Pereira, A.K., M.S. Oliveira, and T.S. Sampaio (2020) ‘Asymmetries of state government social distancing policies in the face of COVID-19: political and technical-administrative aspects’. *Revista de Administração Pública*. 54(4). pp. 678-696.

Peto, J., N. Alwan, K. Godfrey, R. Burgess, D. Hunter, E. Riboli, and P. Romer (2020) ‘Universal weekly testing as the UK COVID-19 lockdown exit strategy’. *The Lancet*. 395(10234). pp. 1420-1421.

Phillips, T. and F. Maisonnave (2020) ‘'Utter disaster': Manaus fills mass graves as Covid-19 hits the Amazon’. *The Guardian*. April 30. https://www.theguardian.com/world/2020/apr/30/brazil-manaus-coronavirus-mass-graves (last accessed on 10 August 2021).

Pickles, J. (2020) ‘Sociality of hate: The transmission of victimization of LGBT+ people through social media’. *International Review of Victimology*. https://doi.org/10.1177/0269758020971060 (last accessed on 10 August 2021).

Pierce, M., H. Hope, T. Ford, S. Hatch, M. Hotopf, A. John, E. Kontopantellis, R. Webb, S. Wessely, S. McManus, and K. Abel (2020) ‘Mental health before and during the COVID-19 pandemic: a

This article is protected by copyright. All rights reserved.

Accepted Article

Accepted Article longitudinal probability sample survey of the UK population'. *The Lancet Psychiatry.* 7(10). pp. 883-892.

Pollock, A., P. Roderick, KK Cheng and B. Pankhania (2020) 'Covid-19: why is the UK government ignoring WHO's advice?' *British Medical Journal*. 368. https://doi.org/10.1136/bmj.m1284 (last accessed on 10 August 2021).

Reid, G. and S. Ritholtz (2020) 'A queer approach to understanding LGBT vulnerability during the COVID-19 pandemic'. *Politics and Gender.* 16. pp. 1101-1109.

Ribeiro, A., J. Kaline and G. Almeida (2021) 'DE QUEM É O CORPO QUE COMPÕE A FORÇA DE TRABALHO? REFLEXÕES SOBRE TRABALHADORAS/ES TRANS NO CONTEXTO DA COVID-19'. *Revista Trabalho Necessári.* 19(38). pp. 152-75.

Ritholtz, S. (2020) 'LGBTQ+ people left out by exclusionary COVID-19 aid practices'. *The New Humanitarian.* June 24. https://www.thenewhumanitarian.org/news-feature/2020/06/24/LGBTQ-gender-coronavirus-discrimination-aid (last accessed on 10 August 2021).

Rodriguez-Morales, A., V. Gallego, *et al*. (2020) 'COVID-19 in Latin America: The implications of the first confirmed case in Brazil'. *Travel Medicine and Infectious Disease*. 35. Article No. 101613. https://doi.org/10.1016/j.tmaid.2020.101613 (last accessed on 10 August 2021).

Singh, A.A. and V.S. Mckleroy (2011) "Just getting out of bed is a revolutionary act": The resilience of transgender people of color who have survived traumatic life events. *Traumatology*. 7(2). pp. 34-44.

Sauer, A.T. and A. Podhora (2013) 'Sexual orientation and gender identity in human rights impact assessment'. *Impact Assessment and Project Appraisal.* 31(2). pp. 135-145.

Ștefăniță, O. and D.M. Buf (2021) 'Hate speech in social media and its effects on the LGBT community: a review of the current research'. *Romanian Journal of Communication and Public Relations*. 23(1). pp. 47-55.

UK Government (2010) *Equality Act.* https://www.legislation.gov.uk/ukpga/2010/15/contents (last accessed on 10 August 2021).

Vickery, J. (2019) 'Using an intersectional approach to advance understanding of homeless persons' vulnerability to disaster'. *Environmental Sociology*. 4(1). pp. 136-147.

##VOTELGBT (2021) *Diagnostico LGBT+ na pandemia*. https://votelgbt.org/pesquisas (last accessed on 10 August 2021).

Wareham, J. (2020) 'Murdered, suffocated and burned alive – 350 transgender people killed In 2020'. *Forbes.* November 11). https://www.forbes.com/sites/jamiewareham/2020/11/11/350-transgender-people-have-been-murdered-in-2020-transgender-day-of-remembrance-list/ (last accessed on 10 August 2021).

Wisner, B., P. Blaikie, T. Cannon, and I. Davis (eds) (2004) *At Risk: Natural hazards, people's vulnerability and disasters 2nd edition*. Routledge, London and New York.

Yamashita, A., C. Gomez, and K. Dombroski (2017) 'Segregation, exclusion and LGBT people in disaster impacted areas: experiences from the Higashinihon Dai - Shinsai (Great East-Japan Disaster)'. *Gender, Place and Culture.* 24(1). pp. 64-71.

This article is protected by copyright. All rights reserved.

Accepted Article

[1] Jadir, gay cisgender man, Brazil (research interview).

[2] The pandemic continued and evolved since our data collection, with new COVID-19 variants and further waves of infections and associated government responses. However, this paper remains focused on insights gained from the first wave study period.

[3] We acknowledge that this sentiment equally applies to other countries too. Our study focuses on the UK and Brazil exclusively due to opportunities to undertake rapid research in these countries afforded by existing research networks and resources. We recommend expanding the scope of this work in the future to explore similar questions in other country contexts.

[4] A pervasive system that assumes and promotes cisgender (identifying with the binary sex (male/female) assigned at birth) and heterosexuality as the norm, privileging them over other gender and sexuality identities and expressions.

[5] We acknowledge transparency is not appropriate for all researchers or at all times. E.g., disclosing gender identity or sexuality in outlining positionality may have negative consequences, such as discrimination.

[6] We acknowledge that mental health challenges may not be experienced or perceived in the same ways in different contexts. Here we consider mental health broadly as a universal indicator of potential concerns, but recognise mental health is not embodied in a universal way, placing some limitations on direct comparisons between cultural contexts.

This article is protected by copyright. All rights reserved.
